# Friday again: Timing and referral patterns of acute radiotherapy in a tertiary supraregional care network

**DOI:** 10.1016/j.ctro.2026.101294

**Published:** 2026-09-17

**Authors:** J. Knoth, E. Suvajac, G. Altorjai, V. Dick, D. Kauer-Dorner, F. Eckert, M. Heilmann, H. Herrmann, M. Hudelist-Venz, S. Konrad, V. Potkrajcic, T. Mrva-Ghukasyan, I.M. Simek, V. Stanisav, A. Sturdza, C. Thiele, L. Ulbrich, C. Waldstein, A. Zaharie, J. Widder, M.P. Schmid

**Affiliations:** Department of Radiation Oncology, Comprehensive Cancer Center, Christian Doppler Laboratory for Image and Knowledge Driven Precision Radiation Oncology, Medical University of Vienna, Vienna, Austria

**Keywords:** Emergency radiotherapy, Oncologic emergencies, Metastatic spinal cord compression, Treatment delay, Referral pathways, Palliative radiotherapy

## Abstract

**Purpose:**

Acute radiotherapy is essential in oncological emergencies, but delays may occur before or after referral to radiation oncology. The primary aim of this study was to characterize timing intervals in a tertiary supraregional care network.

**Methods:**

This retrospective single-centre analysis included patients treated with acute radiotherapy for urgent tumour-related symptoms at our department between 2009 and 2024. Timing intervals from symptom onset to presentation and from presentation to radiotherapy start were analyzed. Referral patterns and weekday distribution were secondary organisational outcomes; symptomatic response and survival were assessed descriptively. All comparative analyses were exploratory and unadjusted.

**Results:**

Overall, 314 patients treated at 349 treatment sites were included. The most frequent indication was metastatic spinal cord compression-related symptoms (260/349; 74.5%). 45.8% of all treatment sites were referred from external institutions. Median time from symptom onset to presentation was 5 days (IQR 2–16.25), whereas median time from presentation to radiotherapy start was 0 days (IQR 0–1). Treatment was initiated on the same day in 218 treatment sites (62.6%) and within one day in 305 (87.6%). Symptomatic response was evaluable for 275 treatment sites, of which 168 (61.1%) showed at least partial improvement. Median survival was 84 days (95% CI: 67–98). Presentations accumulated towards the weekend, with Friday being the most common presentation day.

**Conclusion:**

Acute radiotherapy was initiated rapidly after presentation, whereas a substantially longer interval occurred before presentation to radiation oncology. Improvements should focus on earlier symptom recognition and structured regional emergency pathways.

## Highlights

• Acute radiotherapy was initiated within one day in 87.6% of cases.
• Median time from symptom onset to presentation was 5 days.
• Presentations clustered towards the weekend, with Friday most frequent.
• Reducing pre-referral delays may improve regional emergency pathways.

## 1 Introduction

The clinical relevance of emergency radiotherapy has been demonstrated in a large patterns-of-care study from Germany, Austria and Switzerland, in which emergency radiotherapy accounted for 3.1% of all radiotherapy indications. Reported symptomatic response rates ranged from approximately 50% to 80%, depending on the emergency indication. [1] These data underline that acute radiotherapy is a recurring and clinically relevant part of routine radiation oncology.

Timing is particularly critical in metastatic spinal cord compression (MSCC). Current guideline recommendations emphasise urgent diagnostic work-up, including whole-spine MRI within 24 h in patients with suspected MSCC, or CT-based imaging when MRI is unavailable or contraindicated. [2], [3], [4], [5] Rapid treatment decision-making is essential, not only in patients who are not candidates for surgery. At the same time, functional outcome after radiotherapy is not determined by treatment speed alone. Baseline neurological status, ambulatory function, tumour histology, performance status and the dynamics of neurological deterioration before treatment are known to influence the likelihood of recovery. [6], [7], [8], [9]

Delays may occur at different points along the emergency-care pathway. They may arise before referral because of non-specificity of symptoms, delayed patient presentation, delays in diagnostic work-up, transport logistics, or limited awareness of emergency radiotherapy among referring institutions. Conversely, delays after presentation at the radiation oncology department may reflect internal organisational factors, imaging or treatment-planning availability, weekend or on-call structures and the chosen planning technique. Distinguishing between pre-referral and intradepartmental intervals is therefore clinically relevant when evaluating opportunities for workflow improvement. [1], [10], [11]

The Department of Radiation Oncology at the Medical University of Vienna (MUV) provides acute radiotherapy for a large supraregional referral area in Eastern Austria. The primary objective of this study was to characterize the timing of acute radiotherapy and to determine where time accrued along the emergency-care pathway, with particular focus on the interval from documented symptom onset to presentation at MUV and the interval from presentation to radiotherapy start. Secondary objectives were to describe referral patterns and weekday distribution. Symptomatic response and survival were assessed as descriptive secondary clinical outcomes.

## 2 Methods

### 2.1 Study design and setting

This retrospective single-centre analysis included patients treated with acute radiotherapy for urgent tumour-related symptoms at the MUV between 2009 and 2024. The study was conducted after approval by the responsible ethics committee (EC Nr: 1027/2025). Owing to the retrospective design, no study-related intervention was performed; all treatments were part of routine clinical care.

### 2.2 Patients, treatment sites and data collection

Eligible patients were all who received acute radiotherapy for an oncological emergency or urgent tumour-related symptom requiring immediate or accelerated treatment. Indications included MSCC-related symptoms, superior vena cava syndrome, tumour-related bleeding, pain, threatened vision and other urgent oncological indications. Patients without evaluable treatment documentation, and patients treated within a regular non-emergency workflow, were excluded. The cohort therefore represents patients who ultimately received acute radiotherapy; patients managed primarily by surgery without acute radiotherapy were not captured, and reasons for selecting surgery versus radiotherapy were not systematically available in the retrospective dataset.

Data were retrospectively extracted from institutional clinical documentation systems. Recorded variables included age, referring hospital or institution, underlying tumour histology, emergency indication, lesion location, date of symptom onset where documented, date of presentation at the radiation oncology department, start date of acute radiotherapy, symptomatic response, and survival. The dataset contained 349 treatment-site records from 314 patients; 35 additional treatment-site records represented multiple treatment sites within the same patient.

Lesion location was classified as spinal or non-spinal; for spinal treatment sites, involved regions were documented as cervical, thoracic, lumbar and/or transitional regions where applicable.

### 2.3 Timing intervals, treatment and outcome

Time intervals were calculated from documented calendar dates and included symptom onset to start of acute radiotherapy, symptom onset to presentation at the radiation oncology department, and presentation at the radiation oncology department to start of acute radiotherapy. Symptom onset was defined as the earliest documented onset of the symptom that prompted evaluation and acute radiotherapy for the respective emergency indication. When several symptoms were documented, the onset of the symptom considered clinically related to the treated emergency indication was used. If no sufficiently unambiguous onset date could be derived from the clinical documentation, symptom onset was classified as unknown and the treatment site was excluded from onset-based analyses. Intervals were calculated in days.

Symptomatic response was a descriptive secondary outcome derived from the first evaluable post-treatment clinical documentation and classified as no improvement, partial improvement, complete improvement, or unknown/not evaluable. For selected analyses, response was dichotomised as any improvement, defined as partial or complete improvement, versus no improvement. Response assessment was based on routine clinical documentation and was not collected using a uniform prospective scale.

Survival time was calculated from the start of acute radiotherapy to the documented date of death. The source of death data was the Austrian population register, which is maintained by Statistik Austria. For patients without documented death, the last available follow-up contact was used where available.

### 2.4 Statistics

Statistical analyses were descriptive and exploratory. Categorical variables were reported as absolute and relative frequencies. Continuous variables were described using median and interquartile range (IQR). Group comparisons were performed using the chi-square test, Fisher's exact test, or non-parametric tests, as appropriate. All comparative findings are exploratory and unadjusted. To assess the influence of repeated treatment sites within patients, a patient-level sensitivity analysis excluded the 35 additional site records, yielding one record per patient (*n* = 314). Temporal changes in the primary timing endpoints were explored descriptively for three treatment eras (2009–2014, 2015–2019, and 2020–2024). Survival was estimated using the Kaplan-Meier method. Because of the retrospective design and multiple exploratory comparisons, *p*-values were interpreted descriptively and were not used to infer causality.

## 3 Results

### 3.1 Cohort referral pattern, and lesion location

A total of 314 patients treated at 349 treatment sites were included. Thirty-one patients were treated at multiple sites at the same time, and three patients were treated at different sites at different time points. Median age at the start of acute radiotherapy was 60.6 years (IQR 49.5–69.9), see also Table 1 for patient characteristics. Referrals originated from 36 different hospitals or institutions. The most common referring institution was the MUV itself (*n* = 189 treatment sites; 54.2%). Thus, almost half of the treatment sites originated from external institutions.

**Table 1:** Cohort characteristics. Age, sex, ECOG performance status and primary tumour are reported at patient level (*n* = 314); emergency indications are reported at treatment-site level (*n* = 349). Other primary tumours included diverse gynaecological tumours, GI-tumours, brain tumours, Melanoma, Sarcoma, Thyroid tumours.

Characteristic	Overall
Age, years, median (IQR)	61 (50–70)
**Sex, n (%)**	
Male	177 (56.4%)
Female	137 (43.6%)
ECOG performance status, median (IQR)	2 (1–3), *n* = 43
ECOG 0	5 (1.6%)
ECOG 1	7 (2.2%)
ECOG 2	12 (3.8%)
ECOG 3	10 (3.2%)
ECOG 4	9 (2.9%)
Unknown/not documented	271 (86.3%)

**Primary tumour, n (%) – patient level**	
Lung cancer	70 (22.3%)
Lymphoma	32 (10.2%)
Breast cancer	27 (8.6%)
Prostate cancer	27 (8.6%)
Multiple myeloma	16 (5.1%)
Other	142 (45.2%)

**Indication, n (%) – treatment site level**	
Spinal cord compression	260 (74.5%)
Motor symptoms	31 (11.9% of spinal cord compression)
Sensory symptoms	12 (4.6% of spinal cord compression)
Unknown symptom type	217 (83.5% of spinal cord compression)
Superior vena cava syndrome	27 (7.7%)
Bleeding	19 (5.4%)
Pain	12 (3.4%)
Visual impairment	7 (2.0%)
Other	24 (6.9%)

Most treated lesions were located in the spine. Overall, 264 treatment sites (75.6%) involved spinal sites. When transitional regions were considered, the thoracic spine was involved in 198 of 264 spinal treatment sites (75.0%), the lumbar spine in 78 (29.5%) and the cervical spine in 54 (20.5%). Non-spinal locations accounted for 85 treatment sites (24.4%).

### 3.2 Timing of presentation and treatment

**Fig. 1:**
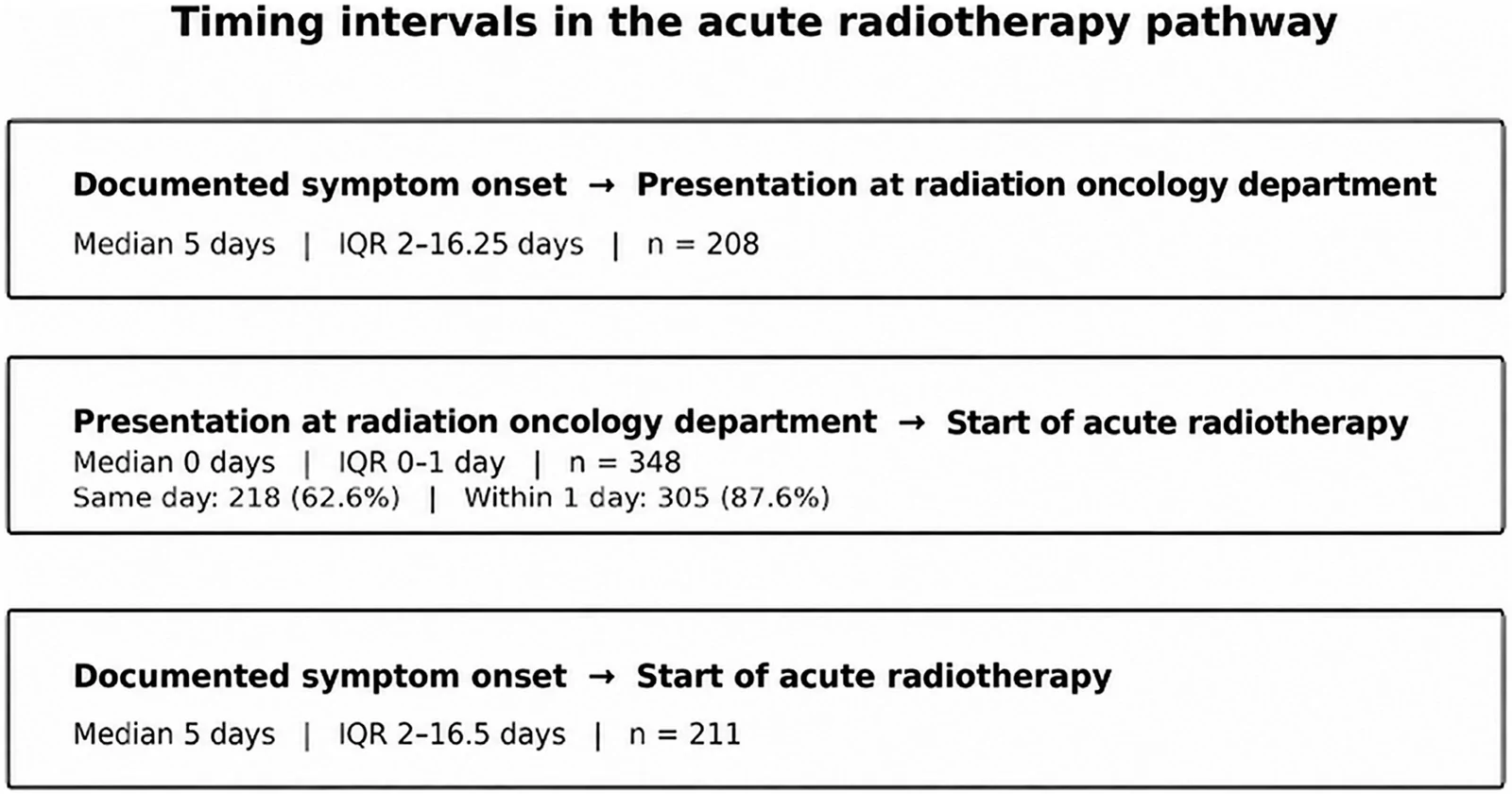
Timing intervals in the acute radiotherapy pathway.

Symptom onset was documented as known in 223 treatment sites (63.9%). The median interval from symptom onset to start of radiotherapy was evaluable in 211 treatment sites and was 5 days (IQR 2–16.5 days), see also Fig. 1. The median interval from symptom onset to presentation at the radiation oncology department was evaluable in 208 treatment sites and was also 5 days (IQR 2–16.25 days). This interval exceeded 14 days in 55 treatment sites (26.4%).

When comparing referrals from MUV with those from external hospitals, the interval from symptom onset to presentation was numerically longer in internal referrals, but this did not reach statistical significance (median 7 days, IQR 2–21 vs. 3 days, IQR 1–13; *p* = 0.069). The interval from symptom onset to start of radiotherapy was longer in internal referrals (median 7 days, IQR 3–23 vs. 4 days, IQR 2–14; *p* = 0.021). This comparative finding is exploratory and unadjusted.

The interval from presentation at the department of radiation oncology to start of acute radiotherapy was documented in 348 treatment sites. The median interval was 0 days (IQR 0–1 day). Treatment was initiated on the same day in 218 treatment sites (62.6%) and within one day in 305 treatment sites (87.6%).

Across the 15-year study period, the median presentation-to-radiotherapy interval remained 0 days (IQR 0–1 day) in each treatment era. Radiotherapy was initiated within one day in 118/143 treatment sites (82.5%) in 2009–2014, 110/121 (90.9%) in 2015–2019, and 77/84 (91.7%) in 2020–2024. The median documented symptom-onset-to-presentation interval was 7 days (IQR 1–23; *n* = 77), 4.5 days (IQR 2–14; *n* = 76), and 4 days (IQR 1.5–10; *n* = 55), respectively. These era analyses were descriptive.

In the patient-level sensitivity analysis excluding 35 additional treatment-site records, the central timing estimates were unchanged: the median documented symptom-onset-to-presentation interval was 5 days (IQR 2–16.25; *n* = 188), and the median presentation-to-radiotherapy interval was 0 days (IQR 0–1; *n* = 313). Treatment started on the same day in 200/313 patients (63.9%) and within one day in 278/313 (88.8%).

The median single fraction dose during acute radiotherapy was 3 Gy (IQR 3–3 Gy). The overall delivered radiation dose, including treatment continuation where applicable, was 30 Gy (IQR 24–39 Gy).

### 3.3 Secondary descriptive outcomes

Symptomatic response, derived from the first evaluable post-treatment clinical documentation, was documented in 275 treatment sites. Partial symptom improvement was observed in 102 treatment sites (37.1%), complete improvement in 66 (24.0%) and no improvement in 107 (38.9%). Thus, 168 of 275 evaluable treatment sites (61.1%) showed at least partial symptomatic response. In 74 treatment sites, response was unknown or not evaluable. Details on response by indication are given in the Appendix.

Survival after radiotherapy was evaluable in all patients. Estimated median survival was 84 days (95% CI: 67–98), Estimated 1-year survival was 17.1% (95% CI: 13.1–21.5).

**Fig. 2:**
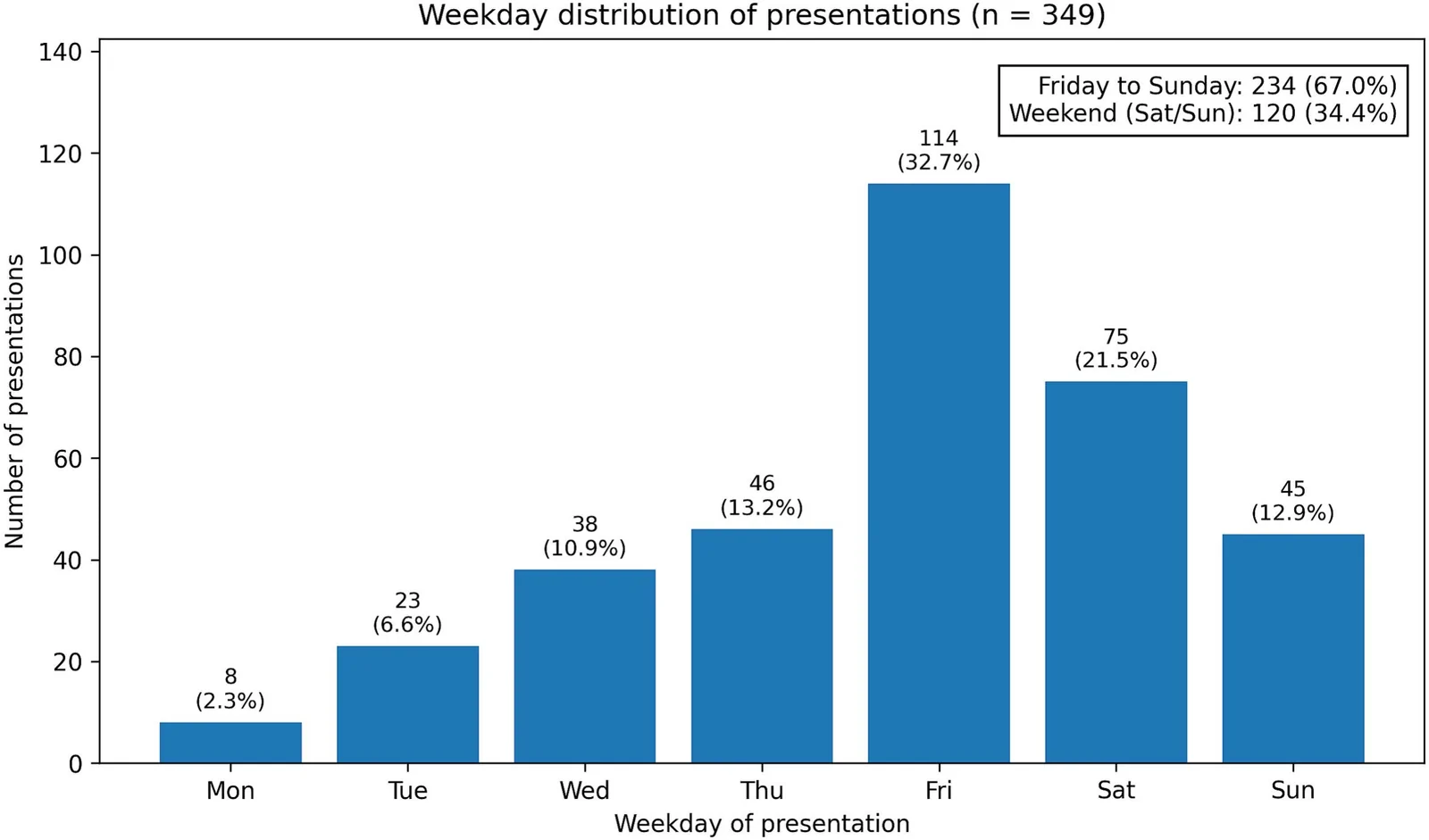
Distribution of referrals per weekday.

Presentation showed an uneven distribution across the week, as given in Fig. 2. Among Monday-to-Friday presentations, partial or complete improvement was documented in 130 of 200 evaluable treatment sites (65.0%), compared with 38 of 75 (50.7%) among Saturday or Sunday presentations (chi-square *p* = 0.042; Fisher's exact *p* = 0.037). Complete improvement was less frequently documented among weekend presentations (12.0% vs. 28.5%).

## 4 Discussion

This retrospective single-centre analysis was designed primarily to determine where time accrued along the acute radiotherapy pathway. The median interval from presentation to treatment start was 0 days, and treatment began within one day in 87.6% of treatment sites. In contrast, the median interval from symptom onset to presentation and from symptom onset to radiotherapy start was 5 days. Similar to the prospective study by Husband, who demonstrated that most delay in MSCC occurred before arrival at the treatment unit, [12] our findings show that the observed pre-presentation interval was substantially longer than the post-presentation interval. Because the individual components of the pre-presentation interval were not separately measured, however, this interval should not be interpreted as a directly quantified or necessarily avoidable referral delay.

This distinction is clinically relevant in a supraregional care network. Almost half of the patients were referred from external institutions. The overall interval from symptom onset to radiotherapy therefore reflects not only radiation oncology availability, but also symptom recognition, diagnostic imaging, interdisciplinary decision-making, transport logistics and awareness of emergency radiotherapy among referring hospitals. Interestingly, externally referred patients did not show longer intervals than internal referrals. The longer symptom-onset-to-radiotherapy interval observed among internal referrals may reflect differences in clinical spectrum, inpatient diagnostic pathways, more complex patients, or delayed recognition of symptoms in already hospitalised patients. The data therefore identify the upstream pathway as a potential target for improvement but do not isolate a specific causal component.

MSCC represented the largest subgroup of indications. Functional outcome in MSCC depends strongly on baseline neurological status, ambulatory function, performance status, tumour histology and the dynamics of symptom progression before treatment. [6], [7], [8], [9], [13] A simple calendar interval from first symptom to radiotherapy may therefore be an insufficient surrogate for clinically relevant urgency. The audit by Levack et al. [14] highlighted that waiting for overt neurological signs may be too late, as pain and radicular symptoms often precede established cord compression. A patient with slowly progressing sensory symptoms over several days may have a better prognosis than a patient with rapidly evolving motor deterioration over a shorter interval. This may explain why the interval from symptom onset to radiotherapy start was not significantly associated with symptomatic response in the present analysis.

The accumulation of presentations towards Friday and the weekend is notable. More than two thirds of treatment sites presented between Friday and Sunday, and Friday was the single most common day of presentation. The present data cannot determine whether this pattern reflects referral timing, diagnostic availability, transport logistics, case mix or other organisational factors. Weekend presentations had a lower documented response rate than Monday-to-Friday presentations. This finding is hypothesis-generating and unadjusted. The data do not prove that weekend care itself leads to inferior outcomes. Nevertheless, the finding is relevant because emergency radiotherapy depends on continuously functioning regional pathways, not only on internal on-call availability.

The long observation period from 2009 to 2024 encompassed changes in imaging, radiotherapy technology and clinical practice. In the descriptive era analysis, the median post-presentation interval remained 0 days in all three eras, while treatment within one day increased from 82.5% in 2009–2014 to more than 90% in the two later eras. The median documented symptom-onset-to-presentation interval decreased descriptively from 7 days in 2009–2014 to approximately 4–4.5 days thereafter. These findings suggest that the principal distinction between a longer pre-presentation and short post-presentation interval was not confined to a single treatment era; nevertheless, temporal changes in case mix, documentation and regional pathways remain potential sources of confounding.

A practical option to shorten preparation time in selected emergencies is the use of already available diagnostic CT data for initial treatment planning, particularly when patients are transferred with recent imaging. [11] Careful selection is required because diagnostic CT differs from dedicated planning CT in positioning, immobilisation, reference marks, couch geometry and HU calibration. This strategy appears most suitable for robust emergency plans, whereas complex highly modulated treatments or situations with relevant anatomical changes may still require dedicated simulation or early replanning.

The clinical implications of the present analysis concern the regional pathway rather than a single causal bottleneck. For patients with suspected MSCC or other time-critical oncological emergencies, improvement efforts should focus on earlier symptom recognition, low-threshold radiation oncology consultation, rapid imaging, standardised referral criteria, and reliable transport and communication pathways between referring institutions and the radiation oncology department. National audit data indicate that timely MRI can be achieved, but that multidisciplinary decision-making, particularly surgical assessment in appropriate patients, remains a vulnerable point in MSCC pathways. [15] Educational initiatives, structured referral algorithms and shared emergency pathways or hotlines [16] could help ensure that patients are referred before severe or rapidly progressive deficits occur.

The study has several limitations. First, it is retrospective and single-centre, with heterogeneous documentation over a 15-year study period. Although imaging and radiotherapy techniques evolved during the study period, no major formal change in the institutional emergency radiotherapy referral or on-call pathway occurred. Second, symptom onset was unavailable, approximate or temporally inconsistent in a substantial proportion of treatment sites; exact onset-based analyses therefore represent complete-case analyses and may be subject to documentation bias. Third, the primary dataset was structured by treatment site and included repeated sites within individual patients. Although the patient-level sensitivity analysis yielded comparable timing estimates, correlation between multiple treatment episodes cannot be fully modelled retrospectively. Fourth, the cohort included heterogeneous emergency indications with different urgency, treatment objectives and expected responses; symptomatic response was derived from routine documentation and not assessed using uniform prospective scales. This is particularly relevant for MSCC, where motor function, ambulation, sensory deficits, and bladder or bowel function were not consistently documented in a standardised manner. Fifth, the MSCC cohort represents patients selected for radiotherapy; surgical eligibility and reasons for treatment selection were not systematically available. Finally, changes in imaging, radiotherapy practice, case mix and regional pathways over time may have influenced timing. All comparative findings should therefore be interpreted as exploratory and not as evidence of causality.

Despite these limitations, the study provides a clinically relevant real-world description of acute radiotherapy in a large tertiary care network. It demonstrates that same-day radiotherapy after presentation is feasible in routine practice, but also shows that the clinically decisive delay may occur before referral. This shifts the focus of quality improvement from the radiation oncology department alone to the entire regional emergency-care pathway, while acknowledging that the present data do not quantify the relative contribution of individual organisational, tumour-related or patient-related factors.

In conclusion, acute radiotherapy for oncological emergencies was initiated very rapidly after presentation at a tertiary radiation oncology department, largely meeting guideline-based expectations for timely treatment after decision-making. In contrast, the substantially longer interval occurred before presentation to radiation oncology. These findings suggest that upstream components of the regional emergency-care pathway may represent important targets for quality improvement. However, the present retrospective analysis cannot determine the relative contributions of organisational, tumour-related and patient-related factors to this interval or establish causal relationships with clinical outcomes. Future improvement efforts should therefore address symptom recognition, diagnostic work-up, interdisciplinary referral and communication across the regional emergency-care pathway.

## Declaration of generative AI and AI-assisted technologies in the manuscript preparation process

During the preparation of this work the authors used ChatGPT 5.5 in order to improve grammar and text fluency. After using this tool, the authors reviewed and edited the content as needed and take full responsibility for the content of the published article.

## Ethics declaration

The authors have NOT obtained informed consent from participants or their legal representatives. This was a retrospective study.

This study was performed in compliance with relevant laws, regulatory frameworks and guidelines where the research took place. This study was approved by the Medical University of Vienna. (Approval No. 1027/2025)

## Declaration of competing interest

The authors declare that they have no known competing financial interests or personal relationships that could have appeared to influence the work reported in this paper. The financial support by the Austrian Federal Ministry of Labour and Economy, the National Foundation for Research, Technology and Development and the Christian Doppler Research Association is gratefully acknowledged.
